# Supplementary material for: Are Sexual Assaults Related to Functional Somatic Disorders? A Cross-Sectional Study
Source: Int J Environ Res Public Health. 2023 Oct 20;20(20):6947. doi: 10.3390/ijerph20206947 (PMC10606494; doi:10.3390/ijerph20206947)
Supplement: Supplementary file 1 [file ijerph-20-06947-s001.zip › ijerph-2578613-supplementary.pdf]

Supplementary Digital Files: Are Sexual Assaults related to Functional Somatic Disorders? A cross-sectional study

Figure S1: Flow chart of participation from the DanFunD population sample

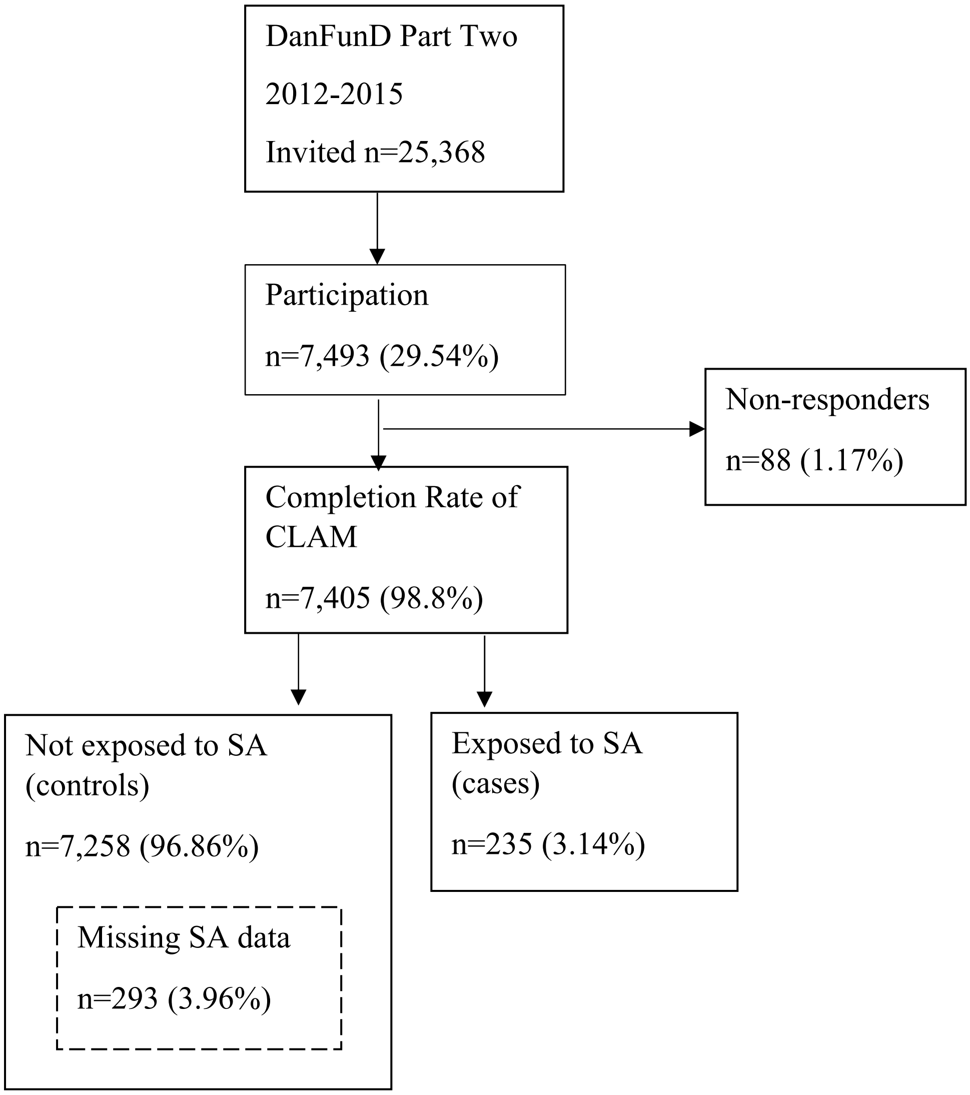

**Table S1, Abbreviations**	
Abbreviations	Description
SA	Sexual Assault
FSD	Functional Somatic Disorder
FSS	Functional Somatic Syndromes
CWP	Chronic Widespread Pain
IB	Irritable Bowel
CF	Chronic Fatigue
MCS	Multiple Chemical Sensitivity
WAD	Whiplash-Associated Disorder
BDS	Bodily Distress Syndrome
PTSD	Post-traumatic Stress Disorder
DanFunD	The Danish Study of Functional Disorders
ISTDP	Intensive Short-Term Dynamic Psychotherapy
EAET	Emotional Awareness and Expression Therapy
CLAM	Cumulative Lifetime Adversity Measure
SCL-8	Symptom checklist, derived from the 90-item SCL-90-R
CI	Confidence interval
RR	Risk Ratio
SSD	Somatic Symptom Disorder

**Table S2**	**Population Characteristics, women**				
**Variables** (Measure)	**Categories**	**Type of assault**			
		**Total Population** *n* = 4,037	**Exposed to Sexual Assault** *n* = 209	Subdivisions on Sexual Assault	
				Rape *n* = 126	Groping *n* = 155
Age at inclusion: Median (IQR)	Baseline	51.6 (43-62)	50.2 (42-61)	51.5 (44-62)	49.9 (42-60)
Age at exposure of SA % (n)	<5 years		11.0 (23)	6.3 (8)	14.8 (23)
	6-11 years		29.2 (61)	18.3 (23)	33.5 (52)
	12-18 years		39.2 (82)	38.1 (48)	37.4 (58)
	>18 years		20.6 (43)	37.3 (47)	14.9 (22)
Vocational training % (n)	No education	11.4 (459)	12.0 (25)	17.5 (16)	14.8 (18)
	<3 years	20.7 (835)	18.7 (39)	20.1 (26)	21.5 (32)
	3-4 years	43.5 (1757)	35.9 (75)	38.9 (43)	39.6 (54)
	>4 years	21.4 (864)	29.2 (61)	16.8 (36)	16.8 (44)
	Missing 3.0 (122)				
Adverse Childhood -environment % (n)	*Missing 1.3 (53)*	6.4 (260)	9.6 (20)	8.7 (11)	11.6 (18)
Physical abuse % (n)	*Missing 1.2 (47)*	12.3 (496)	50.7 (106)	60.3 (76)	54.2 (84)
Emotional abuse % (n)	*Missing (3.7 (148)*	7.7 (311)	37.3 (78)	45.2 (57)	38.7 (60)
Abuse (physical and/or emotional) % (n)	*Missing 3.7 (148)*	15.8 (636)	61.2 (128)	69.1 (87)	65.8 (102)
Social Status Median (IQR)		7 (6-8)	6n (5-7)	6 (5-7)	6 (5-7)
Emotional distress % (n)	*Missing 1.5 (60)*	6.0 (241)	11.5 (24)	8.7 (13)	10.1 (15)
Health anxiety % (N)	*Missing 0.5 (39)*	6.2 (251)	11.5 (24)	13.5 (17)	12.3 (19)
FSD % (n)	Single-organ	19.3 (778)	31.6 (66)	34.1 (43)	29.7 (46)
	Missing 2.2 (90)				
	Multi-organ	1.6 (64)	5.7 (12)	6.4 (8)	7.1 (11)
	Missing 20.0 (804)				

FSS % (n)	CWP	6.4 (257)	9.1 (19)	10.3 (13)	10.3 (16)
	Missing 1.2 (48)				
	IB	4.9 (198)	10.1 (21)	11.9 (15)	9.0 (14)
	Missing 2.1 (85)				
	CF	11.7 (473)	23.9 (50)	27.8 (35)	27.1 (42)
	Missing 1.3 (53)				
	MCS	2.6 (104)	7.2 (15)	7.9 (10)	6.5 (10)
	Missing 1.4 (55)				
	WAD	1.8 (71)	4.3 (9)	4.8 (6)	3.9 (6)
	Missing 2.0 (82)				

Table S2: SA: sexual Assault; FSD: functional somatic disorder; FSS: functional somatic syndrome; CWP: chronic widespread pain: IB: irritable bowel; CF: chronic fatigue; MCS: multiple chemical sensitivity; WAD: whiplash-associated disorder; IQR: Interquartile range.

Individuals missing answers on SA items: *n* = 138

Table S3	Characteristics: Missing data of SA	
**Variables** (Measure)	**Categories**	**Missing SA-cases *n* = 293**
Age at inclusion: Median (IQR)	Baseline	58 (48-66)
Sex % (*n*)	Male	52.90 (155)
	Female	47.10 (138)
Vocational training % (*n*)	No education	11.95 (35)
	<3 years	16.04 (47)
	3-4 years	40.27 (18)
	>4 years	26.28 (77)
Upbringing % (*n*)		0 (0)
Social Status Median (IQR)		7 (6-8)
Emotional distress % (*n*)		6.83 (20)
Health anxiety % (*n*)		0 (0)
Physical abuse % (*n*)		0 (0)
Emotional abuse % (*n*)		2.13 (5)
Abuse % (*n*)		2.13 (5)
FSD % (*n*)	Single-organ	3.63 (46)
	Multi-organ	4.76 (4)
FSS % (*n*)	CWP	5.46 (16)
	IB	3.41 (10)
	CF	8.53 (25)
	MCS	2.05 (6)
	WAD	1.71 (5)

Table S3: Abbreviations: SA: sexual assault; FSD: functional somatic sisorder; FSS: functional somatic syndrome; CWP: chronic widespread pain; IB: irritable bowel; CF: chronic fatigue; MCS: multiple chemical sensitivity; WAD: whiplash-Associated disorder; SD: standard deviation.

**Table S4** **Symptom severity**	**Severity;" The past 12 months, have you been bothered by…" % (N), χ²**				
	**Not at all**	**A bit**	**Somewhat**	**Quite a bit**	**A lot**
			**Cardiopulmonary Symptoms**		
Heartpounding P<0.001					
No SA (6930)	65.3 (4525)	25.7 (1778)	6.4 (440)	2.3 (156)	0.5 (31)
SA (235)	51.9 (122)	31.5 (74)	9.4 (22)	6.0 (14)	1.9 (3)
Rape (139) **p* >0.0001	52.5 (73)	27.3 (38)	10.1 (14)	8.6 (12)	1.4 (2)
Groping	50.3 (90)	33.0 (59)	10.1 (18)	5.6 (10)	1.1 (2)
Precordinal discomfort P***=0.002***					
No SA (6919)	76.6 (5301)	18.2 (1258)	3.8 (265)	1.2 (83)	0.2 (12)
SA (234)	65.4 (153)	23.5 (55)	6.8 (16)	3.9 (9)	0.4 (1)
Rape (138)	65.2 (90)	23.9 (33)	5.8 (8)	4.4 (6)	0.7 (1)
Groping (178) *p>0.0001	65.2 (116)	23.0 (41)	7.3 (13)	3.9 (7)	0.6 (1)
Breathlessness without extertion P<0.001					
No SA (6926)	73.7 (5102)	18.5 (1281)	5.5 (380)	2.0 (136)	0.4 (27)
SA (234)	58.1 (136)	25.6 (60)	9.4 (22)	4.3 (10)	2.6 (6)
Rape (139)	56.8 (79)	23.7 (33)	12.2 (17)	3.6 (5)	3.6 (5)
Groping (178)	55.6 (99)	27.5 (49)	9.00 (16)	5.1 (9)	2.8 (5)
Hyperventilation P<0.001					
No SA (6922)	90.2 (6240)	7.1 (492)	2.1 (144)	0.5 (37)	0.1 (9)
SA (233)	77.3 (180)	14.2 (33)	6.4 (15)	1.7 (4)	0.4 (1)
Rape (138)	72.5 (100)	17.4 (24)	7.3 (10)	2.2 (3)	0.7 (1)
Groping (177)	78.5 (139)	12.4 (22)	6.8 (12)	2.3 (4)	-
Hot or cold sweats P<0.001					
No SA (6910)	67.6 (4672)	22.1 (1526)	6.5 (449)	3.1 (217)	0.7 (46)
SA (233)	53.2 (124)	23.6 (55)	14.6 (34)	7.7 (18)	0.9 (2)
Rape (137)	53.3 (73)	24.1 (33)	13.1 (18)	8.0 (11)	1.5 (2)
Groping (177)	50.3 (89)	28.3 (50)	15.3 (27)	6.2 (11)	-
Dry Mouth P<0.001					
No SA (6912)	71.9 (4972)	19.6 (1356)	5.0 (338)	2.8 (190)	0.8 (56)
SA (235)	56.6 (133)	31.9 (75)	5.5 (13)	3.8 (9)	2.1 (5)
Rape (139)	57.6 (80)	29.5 (41)	4.3 (6)	5.8 (8)	2.9 (4)
Groping (179)	52.5 (94)	34.1 (61)	5.6 (10)	5.0 (9)	2.8 (5)
			**Gastrointestinal symptoms**		
Frequent loose bowel movements P=0.002					
No SA (6924)	55.1 (3814)	31.1 (2154)	9.0 (623)	4.0 (276)	0.8 (57)
SA (234)	47.0 (110)	32.9 (77)	8.6 (20)	9.8 (23)	1.7 (4)
Rape (138)	43.5 (60)	33.3 (46)	10.9 (15)	10.9 (15)	1.5 (2)
Groping (178) *p=0.003	47.2 (84)	32.6 (58)	8.4 (15)	10.1 (18)	1.7 (3)
Abdominal pain P<0.001					
No SA (6897)	64.2 (4429)	26.6 (1834)	6.2 (430)	2.5 (171)	0.5 (33)

SA (233)	45.9 (107)	33.9 (79)	12.0 (28)	7.7 (18)	0.4 (1)
Rape (139)	44.6 (62)	33.1 (46)	12.2 (17)	9.4 (13)	0.7 (1)
Groping (177)	47.5 (84)	33.9 (60)	11.3 (20)	7.3 (13)	-
Feeling bloated P<0.001					
No SA (6917)	49.8 (3445)	33.5 (2317)	10.3 (715)	5.2 (360)	1.2 (80)
SA (235)	28.9 (68)	38.7 (91)	17.9 (42)	11.5 (27)	3.0 (7)
Rape (139)	26.6 (37)	38.9 (54)	18.0 (25)	12.2 (17)	4.3 (6)
Groping (179)	29.1 (52)	40.2 (72)	17.9 (32)	10.1 (18)	2.8 (5)
Diarrhoea P<0.001					
No SA (6921)	73.2 (5066)	21.8 (1506)	3.6 (246)	1.1 (74)	0.4 (29)
SA (235)	60.4 (142)	27.2 (64)	8.1 (19)	3.8 (9)	0.4 (1)
Rape (139)	58.3 (81)	28.1 (39)	9.4 (13)	4.3 (6)	-
Groping (179)	60.9 (109)	27.9 (50)	6.7 (12)	3.9 (7)	0.6 (1)
Regurgitations P<0.001					
No SA (6895)	64.8 (4469)	25.4 (1753)	6.5 (450)	2.6 (178)	0.7 (45)
SA (233)	53.2 (124)	33.5 (78)	8.6 (20)	4.7 (11)	-
Rape (138)	48.6 (67)	34.1 (47)	11.6 (16)	5.8 (8)	
Groping (178)	54.5 (97)	33.2 (59)	8.4 (15)	3.9 (7)	
Nausea P<0.001					
No SA (6935)	79.3 (5499)	16.9 (1172)	2.7 (189)	0.9 (64)	0.2 (11)
SA (235)	62.1 (146)	25.1 (59)	8.1 (19)	3.8 (9)	0.9 (2)
Rape (139)	59.0 (82)	26.6 (37)	8.6 (12)	4.3 (6)	1.4 (2)
Groping (179)	62.0 (111)	25.1 (45)	8.9 (16)	3.4 (6)	0.6 (1)
Burning sensation in upper part of stomach P<0.001					
No SA (6934)	76.8 (5328)	15.7 (1089)	4.9 (341)	2.1 (147)	0.4 (29)
SA (232)	64.7 (150)	21.1 (49)	7.8 (18)	5.6 (13)	0.9 (2)
Rape (136)	60.3 (82)	22.1 (30)	9.6 (13)	6.6 (9)	1.5 (2)
Groping (179)	65.4 (117)	21.2 (38)	7.8 (14)	5.0 (9)	0.6 (1)
**Musculoskeletal symptoms**					
Pains in arms or legs P<0.001					
No SA (6925)	49.4 (3421)	26.9 (1865)	13.0 (902)	8.1 (565)	2.5 (172)
SA (235)	33.6 (79)	23.0 (54)	22.1 (52)	14.8 (34)	6.8 (16)
Rape (139)	30.2 (42)	22.3 (31)	20.9 (29)	17.3 (24)	9.4 (13)
Groping (179)	34.1 (61)	23.5 (42)	20.7 (37)	15.1 (27)	6.7 (12)
Muscular aches or pain P<0.001					
No SA (6907)	37.6 (2599)	38.0 (2622)	14.7 (1014)	8.0 (549)	1.8 (123)
SA (234)	28.2 (66)	30.8 (72)	21.4 (50)	13.7 (32)	6.0 (14)
Rape (139)	24.5 (34)	30.2 (42)	23.0 (32)	13.7 (19)	8.6 (12)
Groping (179)	29.2 (52)	29.8 (53)	20.2 (36)	14.6 (26)	6.2 (11)
Pains in the joints P<0.001					
No SA (6880)	44.0 (3024)	30.4 (2088)	14.5 (997)	9.1 (627)	2.1 (144)
SA (231)	32.5 (75)	26.4 (61)	19.1 (44)	14.7 (34)	7.4 (17)
Rape (138)	29.7 (41)	26.8 (37)	18.1 (25)	15.2 (21)	10.1 (14)

Groping (175)	30.3 (53)	28.0 (49)	19.4 (34)	14.9 (26)	7.4 (13)
Feeling of paresis or localized weakness P<0.001					
No SA (6897)	89.2 (6155)	6.7 (459)	2.4 (162)	1.3 (87)	0.5 (34)
SA (229)	81.2 (186)	9.2 (21)	5.7 (13)	2.2 (5)	1.8 (4)
Rape (134)	76.1(102)	9.7 (13)	8.2 (11)	3.0 (4)	3.0 (4)
Groping (175) ***P***=0.003	80.6 (141)	10.3 (18)	5.1 (9)	2.3 (4)	1.7 (3)
Backache P<0.001					
No SA (6905)	39.6 (2737)	33.9 (2342)	15.3 (1059)	8.4 (583)	2.7 (184)
SA (232)	31.5 (73)	33.2 (77)	14.7 (34)	15.1 (35)	5.6 (13)
Rape (137) *P*=0.005	32.1 (44)	33.6 (46)	12.4 (17)	16.1 (22)	5.8 (8)
Groping (176) *P*=0.001	31.8 (56)	35.2 (62)	11.9 (21)	13.6 (24)	7.4 (13)
Pain moving from one place to another P<0.001					
No SA (6904)	83.0 (5728)	10.1 (694)	4.1 (284)	2.0 (139)	0.9 (59)
SA (233)	72.5 (169)	15.9 (37)	5.6 (13)	3.9 (9)	2.1 (5)
Rape (139)	69.1 (96)	16.6 (23)	7.2 (10)	3.6 (5)	3.6 (5)
Groping (179) *P*=0.007	74.0 (131)	13.6 (24)	6.2 (11)	3.4 (6)	2.8 (5)
Unpleasant numbness or tingling sensations P<0.001					
No SA (6910)	83.5 (5771)	10.2 (706)	3.6 (248)	2.0 (135)	0.7 (50)
SA (235)	69.8 (164)	14.0 (33)	6.8 (16)	8.0 (19)	1.3 (3)
Rape (139)	71.2 (99)	11.5 (16)	7.2 (10)	8.6 (12)	1.4 (2)
Groping (179)	66.5 (119)	16.2 (29)	7.3 (13)	8.9 (16)	1.1 (2)
**General Symptoms**					
Concentration difficulties P<0.001					
No SA (6945)	55.1 (3824)	32.6 (2262)	8.4 (585)	3.0 (211)	0.9 (63)
SA (235)	30.6 (72)	35.3 (83)	21.3 (50)	8.5 (20)	4.3 (10)
Rape (139)	28.1 (39)	35.3 (49)	21.6 (30)	12.2 (17)	2.9 (4)
Groping (179)	29.1 (52)	36.9 (66)	20.7 (37)	8.4 (15)	5.0 (9)
Excessive fatigue P<0.001					
No SA (6949)	30.3 (2102)	42.1 (2930)	16.5 (3)	8.4 (586)	2.7 (185)
SA (235)	18.3 (43)	29.4 (69)	28.5 (67)	16.6 (39)	7.2 (17)
Rape (139)	15.8 (22)	30.2 (42)	25.2 (35)	21.6 (30)	7.2 (10)
Groping (179)	19.6 (35)	27.4 (49)	27.4 (49)	16.8 (30)	8.9 (16)
Headache P<0.001					
No SA (6940)	53.5 (3713)	32.2 (2233)	8.9 (619)	4.1 (283)	1.3 (92)
SA (235)	34.9 (82)	37.0 (87)	15.3 (36)	9.4 (22)	3.4 (8)
Rape (139)	34.5 (48)	36.0 (50)	14.4 (20)	11.0 (15)	4.3 (6)
Groping (179)	32.4 (58)	39.7 (71)	14.0 (25)	10.1 (18)	3.9 (7)
Impairment of memory P<0.001					
No SA (6949)	56.7 (3942)	31.6 (2193)	7.8 (541)	3.0 (205)	1.0 (68)
SA (235)	34.8 (81)	37.0 (87)	12.8 (30)	11.1 (26)	4.7 (11)
Rape (139)	30.2 (42)	39.6 (55)	11.5 (16)	13.7 (19)	5.0 (7)
Groping (179)	34.6 (62)	35.2 (63)	12.9 (23)	11.7 (21)	5.6 (10)

Dizzines P<0.001					
No SA (6942)	72.9 (5061)	20.4 (1418)	4.5 (310)	1.9 (132)	0.3 (21)
SA (235)	54.9 (129)	31.5 (74)	9.8 (23)	3.0 (7)	0.9 (2)
Rape (139)	48.2 (67)	37.4 (52)	11.5 (16)	2.9 (4)	-
Groping (179)	55.3 (99)	30.7 (55)	8.9 (16)	3.9 (7)	1.1 (2)

Table S5	Associations between FSD and sexual assault divided into rape and groping and both																	
**FSD Cases**	**Crude**									**Adjusted***								
	**Groping (n=96)**			**Rape (n=56)**			**Groping and Rape (n=83)**			**Groping (n=96)**			**Rape (n=56)**			**Groping and Rape (n=83)**		
	RR	95% CI	P-Value	RR	95% CI	P-Value	RR	95%CI	P-Value	RR	95% CI	P-Value	RR	95% CI	P-Value	RR	95% CI	P-Value
Single-organ	1.68	1.18-2.37	0.004	2.33	1.64-3.33	>0.0001	2.31	1.70-3.14	>0.001	1.24	0.85-1.81	0.270	2.02	1.38-2.95	>0.0001	1.50	1.07-2.09	0.018
Multi-organ	4.87	1.82-12.99	0.002	2.43	0.34-17.06	0.371	12.52	6.31-24.82	>0.0001	1.44	0.36-5.71	0.607	1.92	0.28-13.34	0.508	6.26	3.25-12.06	>0.0001
**FSS Cases**																		
CWP	1.41	0.65-3.09	0.388	1.21	0.40-3.66	0.736	3.03	1.73-5.31	>0.0001	1.67	0.57-2.39	0.673	1.00	0.34-3.00	0.995	1.53	0.84-2.78	0.163
IB	1.80	0.82-3.94	0.142	3.67	1.82-7.41	>0.0001	3.28	1.75-6.15	>0.001	1.16	0.49-2.75	0.729	2.92	1.45-5.88	0.003	2.36	1.26-4.40	0.007
CF	1.76	1.10-2.82	0.018	1.61	0.84-3.07	0.148	4.08	3.03-5.48	>0.001	1.29	0.80-2.10	0.299	1.46	0.78-2.72	0.234	2.50	1.87-3.34	>0.0001
MCS	2.69	1.13-6.42	0.026	4.61	1.97-10.83	<0.0001	4.47	2.16-9.25	>0.0001	2.07	0.78-5.49	0.144	4.56	1.93-10.79	0.001	3.16	1.42-7.00	0.005
WAD-	2.74	1.03-7.30	0.043	3.56	1.16-10.86	0.026	3.26	1.23-8.63	0.017	1.98	0.63-6.15	0.238	3.55	1.16-10.91	0.027	2.76	(1.02-7.43)	0.044

*Table S5: *Adjusted for age at inclusion, sex, social status, vocational training, adverse childhood environment. Abbreviations: RR: risk ratio; SA: sexual assault; FSD: functional somatic disorders; BDS: bodily distress syndrome; FSS: functional somatic syndrome; CWP: chronic widespread pain; IB: irritable bowel; CF: chronic fatigue; MCS: multiple chemical sensitivity; WAD: whiplash-associated disorder.*
